# Handy Book of Medical Progress

**Published:** 1899-11

**Authors:** 


### Book Reviews.

HANDY BOOK OF MEDICAL PROGRESS. A lexicon of the recent advances in medical science. By CHARLES WARRENNE ALLEN, M. D., Consulting Dermatologist to the Randall's Island Hospitals, and JACOB SOBEL, A. B., M. D., Dermatological Assistant at the Good Samaritan Dispensary. New York: William Wood & Co. 1899.

So rapidly do the advances in the various branches of medical science multiply, that one finds it an almost impossible task to keep abreast of the times without the aid of what might be called concentrated literature. In fact the busy practitioner is obliged to fall back upon such publications to keep him informed of what medical science is doing. So many new words are introduced and coined almost daily that find their way into text-books, encyclopedias and dictionaries, that unless he has some key to their solution he is in absolute darkness. For these reasons the authors have been led to compile a volume arranged alphabetically, giving so far as possible the meaning of new terms, and value of new discoveries in the realms of practical medicine.

Great care and skill has been shown in the enumeration of new medicines and only those which have been kindly received by the profession and shown their good qualities have been incorporated. The formulæ of many of the more important compounds are given, as for instance, Schleich's chloroform mixture, ammonal, antiseptin, antikamnia, bromidia, castoria, camphoid and the like. The formulæ of the various staining mixtures in histology, pathology and bacteriology are also given. The different tests, surgical suggestions and names of new symptoms and new diseases, and new instruments are all briefly described.

It is, in short, a most useful adjunct to the medical dictionary and furnishes a means of rapidly and conveniently acquiring information relative to the newer advances.

The paper and print are excellent, the size convenient, all attesting to the good name of the publishing house whence it came.

